# Transient Liquid Phase Bonding of Ti-6Al-4V and Mg-AZ31 Alloys Using Zn Coatings

**DOI:** 10.3390/ma12050769

**Published:** 2019-03-06

**Authors:** Abdulaziz AlHazaa, Ibrahim Alhoweml, Muhammad Ali Shar, Mahmoud Hezam, Hany Sayed Abdo, Hamad AlBrithen

**Affiliations:** 1 Research Chair for Tribology, Surface, and Interface Sciences, Department of Physics and Astronomy, College of Science, King Saud University, P.O. Box 2455, Riyadh 11451, Saudi Arabia; ialhoweml@ksu.edu.sa (I.A.); Brithen@ksu.edu.sa (H.A.); 2 King Abdullah Institute for nanotechnology, King Saud University, P.O. Box 2455, Riyadh 11451, Saudi Arabia; mashar@ksu.edu.sa (M.A.S.); mhezam@ksu.edu.sa (M.H.); 3 Center of Excellence for Research in Engineering Materials (CEREM), King Saud University, P.O. Box-800, Riyadh 11421, Saudi Arabia; habdo@ksu.edu.sa; 4 Mechanical Design and Materials Department, Faculty of Energy Engineering, Aswan University, Aswan 81521, Egypt; 5 National Center for Nanotechnology, King Abdulaziz City for Science and Technology, P.O. Box 6086, Riyadh 11442, Saudi Arabia

**Keywords:** TLP Bonding, Mg alloy, Ti alloy, coatings, Zinc, shear strength

## Abstract

Ti-6Al-4V and Mg-AZ31 were bonded together using the Transient Liquid Phase Bonding Process (TLP) after coating both surfaces with zinc. The zinc coatings were applied using the screen printing process of zinc paste. Successful bonds were obtained in a vacuum furnace at 500 °C and under a uniaxial pressure of 1 MPa using high frequency induction heat sintering furnace (HFIHS). Various bonding times were selected and all gave solid joints. The bonds were successfully achieved at 5, 10, 15, 20, 25, and 30 min. The energy dispersive spectroscopy (EDS) line scan confirmed the diffusion of Zn in both sides but with more diffusion in the Mg side. Diffusion of Mg into the joint region was detected with significant amounts at bonds made for 20 min and above, which indicate that the isothermal solidification was achieved. In addition, Ti and Al from the base alloys were diffused into the joint region. Based on microstructural analysis, the joint mechanism was attributed to the formation of solidified mixture of Mg and Zn at the joint region with a presence of diffused Ti and Al. This conclusion was also supported by structural analysis of the fractured surfaces as well as the analysis across the joint region. The fractured surfaces were analyzed and it was concluded that the fractures occurred within the joint region where ductile fractures were observed. The strength of the joint was evaluated by shear test and found that the maximum shear strength achieved was 30.5 MPa for the bond made at 20 min.

## 1. Introduction

The growing concerns regarding fuel consumption within the aerospace and transportation industries led to the development of fuel-efficient systems to overcome significant engineering challenges. Mg-AZ31 and Ti-6Al-4V alloys are separately used in the automotive industries due to their excellent physical and mechanical properties such as high specific strength, low mass density, and good machinability and workability [1,2,3]. Ti-6Al-4V alloy covers more than 50% of industrial titanium in the market due to its balance between having high specific strength and good corrosion resistance. On the other hand, the Mg-AZ31 alloy is one of the most popular magnesium alloys. These two alloys are increasingly used in similar sectors. For example, in the automotive industry, titanium has been mainly used in high temperatures zones, and high strength requirement areas, such as exhaust systems, suspension springs, valve springs, valves, and connecting rods. Mg alloys are used in steering hanger beam, steering wheels, transmission outer parts, and seat frames. Therefore, fabricating a joint assembly that combines both alloys is of high interest. However, the vast difference between their melting points makes welding them using commercial methods like fusion welding unsuccessful. Moreover, the binary phase diagram of Mg-Ti system expects very limited mutual solubility. The phase diagram also indicates that no intermetallic compound (IMC) could form between the two metals. It was reported that the solubility of Mg in titanium is 1.6 at% at 765 °C where the solubility of Ti in magnesium is 0.12 at% the same temperature [4]. This means magnesium cannot be welded directly to titanium by solid state diffusion bonding under conventional conditions. Bonding various types of alloys was successfully achieved using transient liquid phase bonding (TLP). In this TLP method, the differences in the physical and mechanical properties of the two alloys can be overcome by inserting an interlayer or applying coatings such as between the two mating surfaces prior to the bonding process [5,6,7]. The challenge of the TLP bonding is to choose a suitable interlayer material that can interact with both materials and form a liquid phase at the selected bonding temperature so a higher diffusion rate can be achieved [8,9]. An important effect of forming a liquid phase at the joint region is the disruption of the native oxide layer that usually forms at metallic surfaces especially for light metals like Ti, Mg, and Al. Studies have shown that the formation of a metallic liquid phase during the bonding process disrupts the formation and growth of stable oxide films. Such oxide formation could prevent successful bonding [10,11]. Many successful examples of dissimilar joints produced by TLP bonding were reported in the scientific literature. Those joints were not possible to achieve using a commercial fusion bonding technique including high precision and well localized welding such as laser or electron beam welding. For instance, the Al7075 alloy was bonded to the Ti-6Al-4V alloy using Cu interlayer and Cu coatings with the Sn interlayer [12,13]. Mg-AZ31 was bonded to the 316 austenitic stainless-steel using the Cu interlayer [14]. Al was bonded with Mg using the Ni interlayer [15].

TLP bonding of Mg AZ31 to Ti-6Al-4V were reported in two studies [16,17]. The first study used Ni coatings. Bonding Mg AZ31 to Ti-6Al-4V using Ni coatings resulted in eutectic formation between the Mg AZ31 and Ni at the Mg side, but, at the Ti side, there was no Ni/Ti eutectic formation occurrence. The bond at the Ti side was interpreted as a result of solid-state diffusion, which is a slow process and needs a long bonding time. Moreover, there is a need of much higher temperature to facilitate the inter-diffusion between Ti and Ni, which is higher than the melting point of the Mg alloy [16]. Another study used a combination of Cu coatings and Cu coatings with a Sn interlayer and reported the formation of Sn_5_Ti_6_ and Mg_2_Cu IMC’s at the joint region where the fracture occurs [17]. Research used Spark Plasma Sintering technology to bond magnesium to titanium with various amount of Al impurities in magnesium and found that the joints occurred as a result of Al diffusion into the joint region where Ti_3_Al formed [18].

Generally, for TLP bonding, it is desirable to form solid solutions at the joint region rather IMC’s in order to gain high strength and avoid the formation of cracks at the interfaces between the formed IMC’s and base alloys. Therefore, it will be interesting to fabricate interlayer/coatings that can react with both dissimilar surfaces and form solid solution across the joint region. Zinc seems to be a potential interlayer to bond Mg with Ti alloys since Zn has good solubility in both Mg and Ti. Zn forms eutectic reaction with Ti that could result in forming Zn and Zn_15_Ti at 418 °C where peritectic points were also reported between the two metals at 486 °C [19]. On the other hand, the ternary phase diagram of Mg and Zn shows eutectic point at 340 °C at the Mg rich region and a eutectic point 364 °C at the Zn rich region where the melting point of Zn is 419.6 °C. Zn is considered to be an alloying element for Mg that improves the castability and corrosion behavior of the magnesium alloy (AZ31) [20]. It was reported that, in the range of 375 °C to 575 °C, the activation energy and pre-exponential factor of the impurity diffusion of Zn in Mg is 109.8 kJ/mol and 10^−5^ m^2^/s [21]. More studies showed that Zn diffuses in the Mg matrix faster than many other alloying elements like Al and even faster than the self-diffusion of Mg [22]. 

Zn interlayers were already used to bond Mg to Al and showed significant improvement in bond quality compared to direct bonding of Mg to Al. The shear strength of the bonded aluminum to magnesium was reported to have a maximum value of 83 MPa, which is twice the maximum value of the shear strength produced by direct bonding of aluminum to magnesium [23]. The zinc interlayer was not used before to bond the Ti alloy with dissimilar materials like Mg alloys. Therefore, the aim of this research study is to apply zinc coatings on the mating surfaces in order to facilitate the bonding between Ti-6Al-4V and Mg-AZ31 alloys. Bonding times were chosen as a variable in order to investigate the effect of bonding time on the bond formation and strength of the joints. A bonding temperature of 500 °C was selected because it is above the eutectic temperatures between Mg and Zn on one hand. On the other hand, the phase diagram study of Ti-Zn suggests that solid solution with IMC’s such as TiZn3 and Ti_2_Zn_3_ can be formed at 500 °C [24]. 

## 2. Experimental Procedure

Commercial magnesium (AZ31) and titanium (Ti-6Al-4V) alloys were purchased from Goodfellow (Cambridgeshire, UK) in a form of extruded cylindrical rods. The diameter of the rods was selected to be 10 mm to fit the bonding machine specifications. Each rod was sliced by the Diamond cutter to produce many identical samples of a thickness of about 5 mm. Every sample was grinded starting from 80 grit to 1000 grit using SiC sand paper. Magnesium alloy samples were grinded with acetone instead of water to reduce the corrosion of the surfaces during the grinding process. Afterward, the samples were ultrasonically cleaned in ethanol medium for 20 min and then kept in a desiccator.

### 2.1. Zn Coatings Using the Screen Printing Technique

Additionally, 30 g of zinc micro-powder (Sigma Aldrich, St. Louis, MO, USA) was dispersed in an organic vehicle composed of 6 g of α-terpineol (Acros Organics, Morris Plains, NJ, USA) and 4 g of 10% weight ethyl cellulose (Sigma Aldrich) in ethanol. The mixture was mixed and grinded by a mortar-pestle for 30 min, which was followed by vigorous magnetic stirring for another 30 min. The mixture was homogenized using a rod homogenizer for 30 to 40 min. The resulting paste was deposited on each metallic disc using a screen printing technique [25]. The screen-printing process was performed using a polyester screen mesh of 32T mesh count (80 mesh/inch), 100 µm thread diameter, and a 210 µm mesh opening. The coating process was optimized to produce a thickness of 10 µm on each surface.

### 2.2. Transient Liquid Phase Bonding

**Figure 1:**
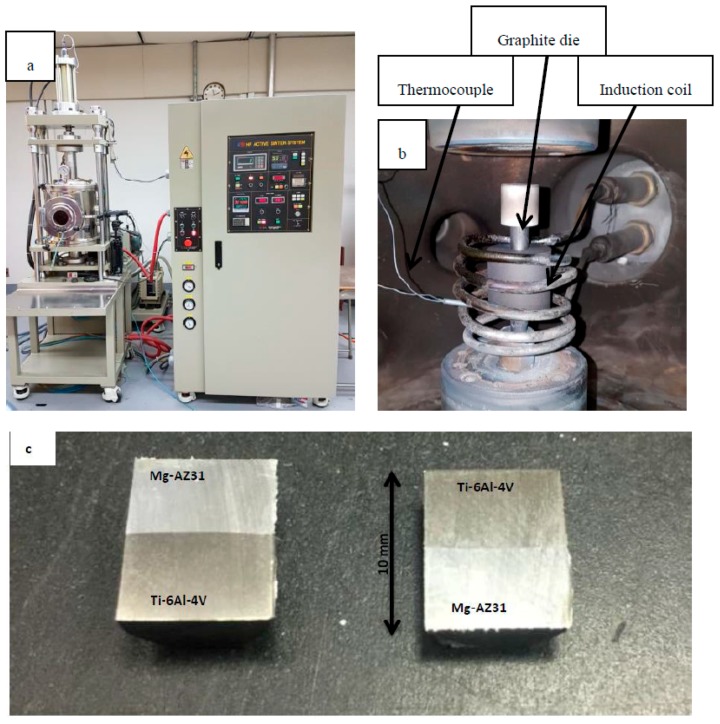
Photos of (**a**) high-frequency induction heating sintering (HFIHS) machine used for bonding experiments. (**b**) Inside look at the vacuum chamber. (**c**) Sample photo of the two pieces of a bond after cutting.

**Figure 2:**
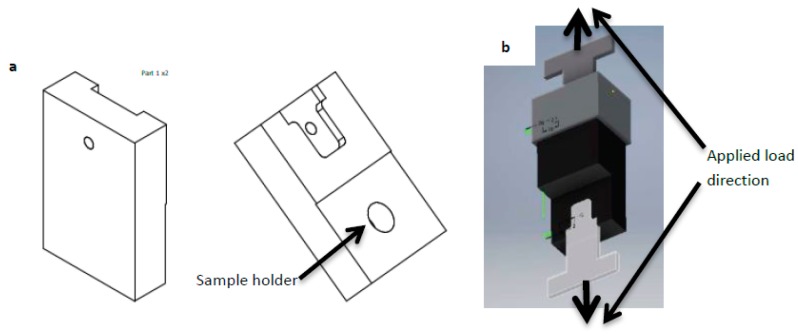
Fixture of the shear testing for bonded samples. (**a**) Back side and front side. (**b**) Full view of the fixture.

Bonding experiments between the Mg-AZ31 alloy and the Ti-6Al-4V alloy were performed in a high frequency induction heating sintering system (HFIHS). Figure 1a shows the HFIHS made by ELTek Co. (Gyeonggi-do, Seol, Korea) where Figure 1b shows the insider image where the bond occurs. The uniaxial pressure was fixed for all experiments to be 1 MPa. Once the vacuum reaches 10^−2^ mbar, a heating rate of 300 °C per minute was applied until reaching a temperature of 500 °C. The holding (bonding) time at 500 °C is considered to be the variable. For each bonding experiment once the bonding time was reached, the bonded sample was cooled down under vacuum to an ambient temperature and then taken out from the chamber. There were two sets of bonded samples. The first set was used for metallographic observations and micro-hardness measurements, and the second set was used for a shear strength test and a fractured surfaces analysis. Therefore, the bonded samples that belong to the first set were cut perpendicular to the joint plane and then mounted in Bakelite powder (Buehler, Lake Bluff, IL, USA) for better handling. The bonded samples were ground to 1200 grit and then they were polished in Al_2_O_3_ suspension down to 1 µm. A JSM-7600F scanning electron microscope (SEM, Joel, Tokyo, Japan) was used to characterize the microstructure and perform line scan and EDS mapping of the joints. X-ray Photoelectron Spectroscopy (PHI VersaProbe XPS Microprobe, Chigasaki, Japan) was used to analyze the interface at the joint line close to the Ti-6Al-4V side. The second set of the samples were fractured using a universal tensile testing machine (Instron 3360, Norwood, MA, USA). A special design for the cylindrical bonded samples was made to fit with the tensile machine in order to measure the shear strength, as shown in Figure 2. SEM and EDS analysis were used to study the fractured surfaces. The X-ray Diffraction (XRD) measurements were carried out using CuKα radiation source (wavelength = 0.154 nm) operated at 45 kV and 40 mA, and the signal was collected using a PIXcel detector (PAnalytical, Almelo, The Netherlands) for 2θ range of 5 to 80° with 0.02° step size to identify the phases present at the fractured surfaces.

## 3. Results and Discussions

### 3.1. Evolution of the Interfacial Layer

**Figure 3:**
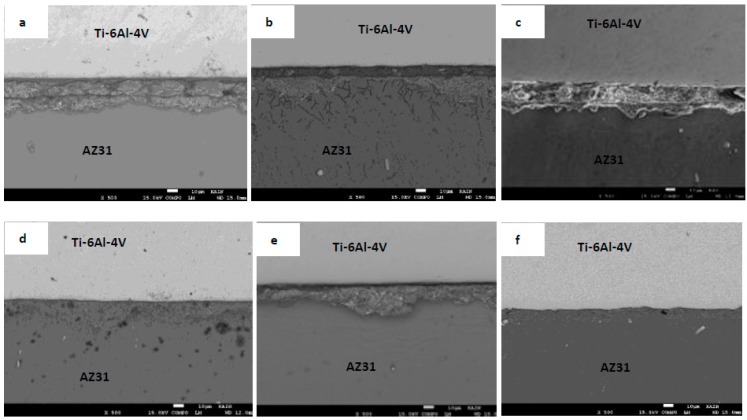
Backscattered SEM images of the joint regions for the bonds made at (**a**) 5, (**b**) 10, (**c**) 15, (**d**) 20, (**e**) 25, and (**f**) 30 min. Scale bar: 10 μm.

**Figure 4:**
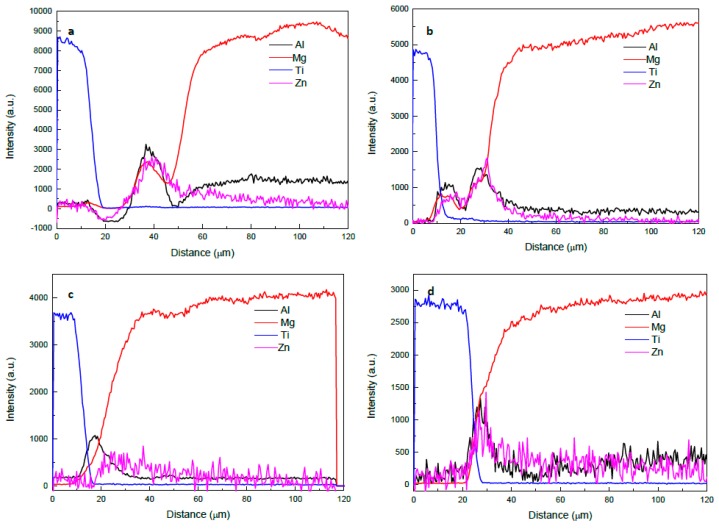
EDS line scans of Mg, Ti, Zn, and Al across the joint region for bonds made at (**a**) 5, (**b**) 15, (**c**) 20, and (**d**) 30 min.

Figure 3 shows SEM micrographs of the joints produced at different bonding times. The width of the joint region remains almost constant in Figure 3a–c, which indicates that the maximum width of the liquid zone was already reached. Therefore, the bonds made at 5, 10, and 15 min are expected to be in the liquid zone homogenization, according to the TLP bonding process [6]. On the other hand, for bonds made at 20, 25, and 30 min shown in Figure 3d–f, the width of the joint region was reduced due to loss of solute by diffusion and the isothermal solidification. By observing the relation between the width of the joint region and the bonding time, it can be concluded that the joint was produced mainly by forming a solidified melt. This means no intermetallic compound (IMC) layer was formed at the joint region. Otherwise, a proportional relation between the joint region width and the bonding time will be observed [12,26,27]. The diffusion rate of zinc in magnesium can be calculated from the frequency factor and activation energy available in the literature [21] to be 1.03 × 10^−5^ m^2^/s. This value is higher than the diffusion rate of Al in Mg at the same temperature [22]. On the other hand, the diffusion coefficient data for zinc in titanium is not available in the literature even though it is expected to be much lower [24]. Since the bonding temperature used is 500 °C and the melting point of Zn is 419.5 °C, the mechanism of bonding is expected to start with complete melting of the Zn coatings, which is followed by diffusion of Zn in Mg and dissolution of Mg in the molten Zn where a eutectic reaction between Mg and Zn occurs at the Mg side of the joint. The dissolution of Ti in the molten Zn and diffusion of Zn in Ti are expected to proceed as well, but with much slower rates. A line scan of Ti, Mg, Zn, and Al was taken across the joint region at various bonding times in order to study the diffusion mechanism for the various elements. Figure 4 shows the EDS line scan across the joint region following the vertical lines that appeared in Figure 3a,c,d,f. From Figure 4, it can be seen that there is a noticeable diffusion of Al from the base alloys into the joint region. A peak of Al is observed for all bonds, which suggests that Al contributes to the joining mechanism either as IMC’s or as dissolved solute in Zn-Mg solid solution. This observation agrees with a recent study that used Spark Plasma Sintering technology to bond Mg with Ti without interlayers and with various Al contents in the Mg [18]. The study showed that Al diffused from the Mg base alloy to the interface forming Ti_3_Al IMC. From Figure 4, it can be seen that the dominant diffusion is the diffusion of Mg into the joint region where Zn was also diffusing away with more into the Mg side. Figure 4c,d show that the Mg line occupied the joint region intersecting with the Ti line. This means that the joint region at bonds made for 20 min and above was rich in magnesium. Isothermal solidification may be reached. On the other hand, the figure shows that Ti diffusion into the joint region is limited.

### 3.2. Morphology and Composition of the Joint Region

**Figure 5:**
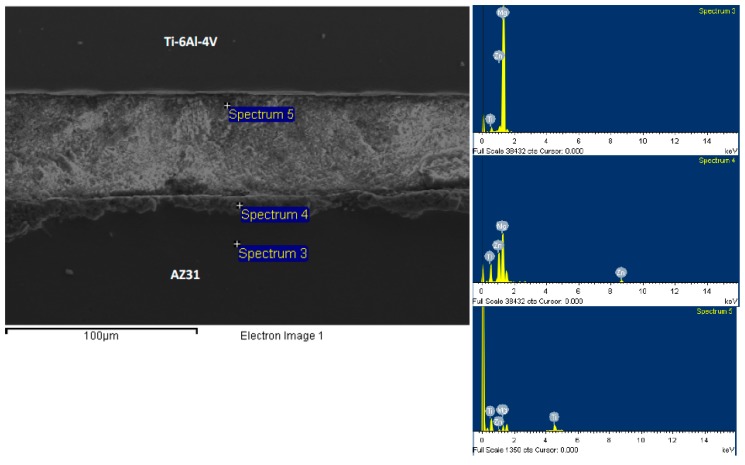
SEM micrograph of the bond made at 5 min and corresponding EDS spot analysis.

**Figure 6:**
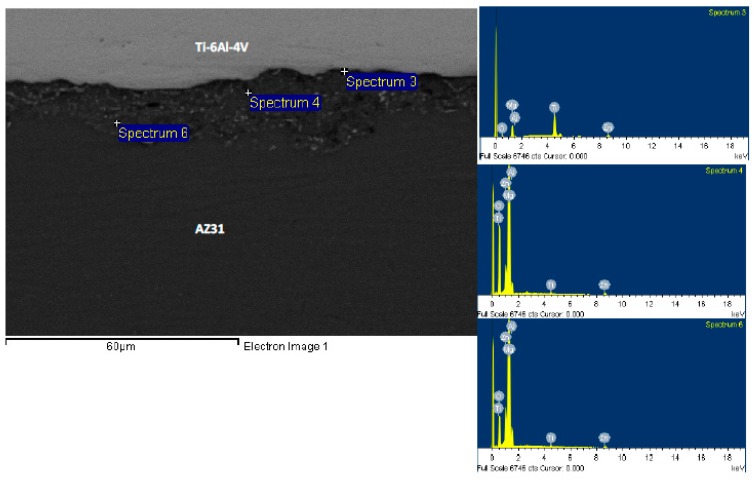
SEM micrograph of the bond made at 30 min and corresponding EDS spot analysis.

In order to investigate the morphology and composition of the joint region and their change with respect to bonding time, we have chosen three bonds for detailed EDS spot analysis. Figure 5 and Figure 6 show EDS spot analysis of various locations in the joint region for bonds made at 5 min and 30 min, respectively. For spectrum 3 that was taken far away from the joint region in the Mg side for the bond made at 5 min, the weight percentages of Mg, Ti, Zn, and Al were measured to be 96.7 wt%, 0.13 wt%, 3.17 wt%, and 2.2 wt%, respectively. The presence of Zn indicates that the diffusion of Zn through the Mg base alloy was noticeable even during the shortest bonding time. The detection of traces of Ti indicates that Ti was able to diffuse into the Mg base alloy regardless of the limited solubility between Ti and Mg. For spectrum 4 in Figure 5, the weight percentages of Mg, Ti, Zn, and Al were measured as 63.1 wt%, 0.53 wt%, 36.4 wt%, and 9.8 wt%, respectively. These values are expected when compared to the measured values in spectrum 3 where more Zn is expected to diffuse away to the Mg base alloy. However, it seems that, for a bond made at 5 min, the remaining Zn at the joint region is excessive. The weight percentage of Mg, Ti, Zn, and Al, which were taken from spectrum 5 were measured to be 28.2 wt%, 61.9 wt%, 9.9 wt%, and 10.5 wt%, respectively. This spot analysis was taken from a joint region that is near the Ti/joint interface with about 10 µm from the Ti side. The measured elemental composition indicates diffusion/dissolution of Ti in the molten Zn, which was originally occupied at the joint region. Furthermore, a noticeable presence of Mg in this region due to the diffusion of Mg in the molten Zn is confirmed. Al was detected in various locations at the joint region and its weight percent was measured to be around 10%. This measurement confirms the diffusion of Al from the base alloy to the joint region. A similar kind of analysis was done with selected spots for the bond made at 30 min, as shown in Figure 6. The EDS analysis for spectrum 3 (near the Ti/joint interface) gave weight percentages of Mg, Ti, Zn, and Al as 18.4 wt%, 72.9 wt%, 1.7 wt%, and 1.1 wt%. With the high percentages of Ti and Mg and low percentage of Zn, the isothermal solidification is expected to be complete. This trend is understood for the TLP process where more Zn diffused away from the joint region. For spectrum 4 (at the joint region), 90.6 wt%, 0.9 wt%, 6.5 wt%, and 2.7 wt% are the percentages of Mg, Ti, Zn, and Al. Lastly, for spectrum 6, which was taken close to the Mg/joint interface, the weight percentages of Mg, Ti, Zn, and Al were measured to be 93.1 wt%, 0.37 wt%, 6.4 wt%, and 4.4 wt%. In comparison with the bond made at 5 min, the joint region for the bond made at 30 min was seen to be occupied with Mg and Ti where less Zn was present. The reduction of the weight percent of Al to be less than 5% compared to the amount detected in the joint region taken from the bond made for 5 min could be attributed to the increase of Ti and Mg percentages. This indicates that the diffusion of Mg and Ti to the joint region is time-dependent where the quantities increase with the increase of bonding time. On the other hand, the diffused quantity of Al in the joint region does not increase much with increasing bonding time. This could be due to the fact that Al is only an alloying element with only 3 wt% in the Mg alloy and 6 wt% in the Ti side. Therefore, the source of Al from the base alloys is limited.

## 4. Analysis of the Fractured Surfaces

**Figure 7:**
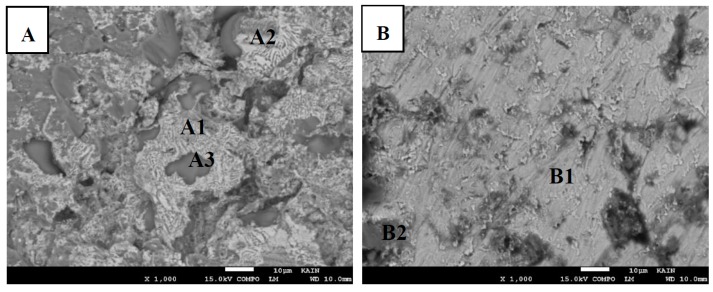
The fractured surfaces of bond made at 5 min. (**A**) Mg side. (**B**) Ti side.

Bonds made at 500 °C with different bonding times were fractured in order to study the morphology and composition of fractured surfaces and, therefore, determine the mechanism and type of fracture. The bonds were fractured using a shear test, which will be discussed in Section 5. Figure 7a,b show the fractured surfaces for bond made at 5 min where Figure 7a represents the Mg fractured surface and Figure 7b represents the Ti fractured surface. Table 1 shows the corresponding elemental composition obtained by EDS spot analysis. In Figure 7a, there are lamellar structure (eutectic like regions) as well as scattered white regions and dark regions in the Mg side. These specific regions were analyzed by EDS. To follow the labeling in the figure, A1 (eutectic) reveals the presence of 46.9 wt% and 47.0 wt% that corresponded to 67.1 at% and 25.0 at% atomic percent for Mg and Zn, respectively. It should be noted that in the Mg-Zn binary phase diagram, the atomic ratio of Zn should be 28.1% in order for the eutectic reaction to occur. Mg and MgZn IMC’s are predicted to form as a result of the eutectic reaction. Therefore, the atomic composition that is corresponding to the lamellar structure A1 (eutectic) can be attributed to eutectic MgZn and Mg phases. The presence of 7.9 at% of Al in this Mg-Zn eutectic region can be explained by the Al-Mg-Zn ternary phase diagram where Al can be dissolved in this eutectic region. The A2 (white) region is richer in Zn compared to A1 (eutectic) region. This region could contain other IMC’s based on Zn and Mg. The percentage of Al in this region is not significantly different from the A1 (eutectic) region. Therefore, no Al based IMC’s is expected to form in the A2 region. The dark region presented by A3 (dark) consists of high quantity of Mg (~94%) with less than 5% of both Zn and Al. This region consists of the Mg-based alloy where most of Zn was diffused away and isothermal solidification in this region took place. In Figure 7b, which is the fractured surface of the Ti for the bond made at 5 min, two distinctive regions were identified by the backscattered SEM and analyzed by EDS spot analysis. The B1 (white) region gave a composition of about 97% of Ti. Therefore, this region represents the Ti surface with less than 4% of Mg, Zn, and Al. The B2 (dark) region consists mainly of Mg with 72.4%. Much less of Zn and Ti were detected in the B2 (dark) region as 7.3% and 4.6%, respectively. The composition of this region is close to the composition of A3 (dark) in the Mg fractured surface. In addition, there is a significant amount of Al (~15.3%) that was detected in this region. The presence of a higher percentage of Al is understood since the solubility of Al in Mg rich phase is high. It is concluded that A3 and B2 are solid solution phases based on rich Mg where B1 is a solid solution based on Ti. Since it is known that the mutual solubility of Ti and Mg is very limited, it is expected that Mg and Ti were both diffused through Zn.

**Figure 8:**
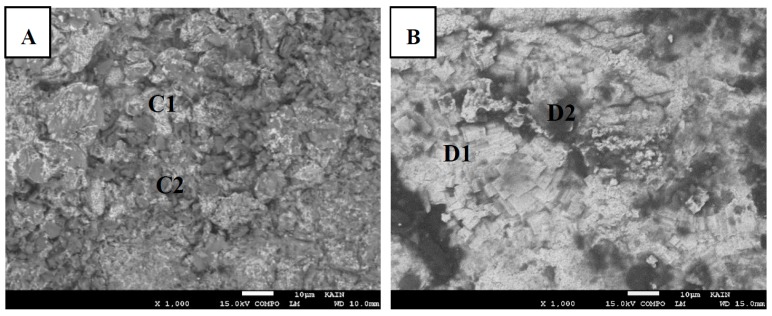
The fractured surfaces of bond made at 20 min. (**A**) Mg side. (**B**) Ti side.

**Figure 9:**
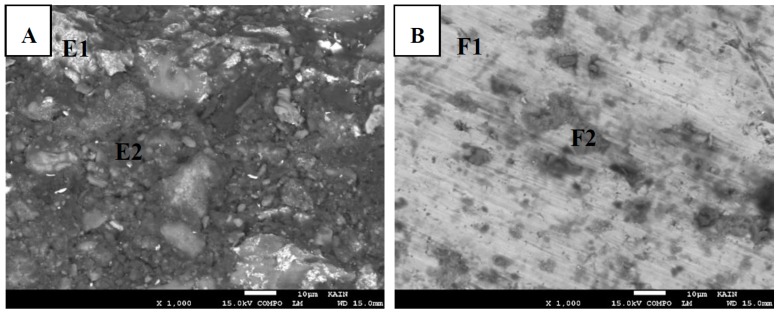
The fractured surfaces of bond made at 30 min. (**A**) Mg side. (**B**) Ti side.

Figure 8a,b show the fractured surfaces for bond made at 20 min where Figure 8a represents the Mg side and Figure 8b represents the Ti side. Table 2 shows the corresponding elemental composition obtained by EDS spot analysis. Less lamellar structure regions are present in Figure 8a compared to Figure 7a. This can be due to more diffusion of Mg into the joint region. Therefore, the solid solution based on Mg became the major structure, as predicted from the Mg-Zn phase diagram. EDS spot analysis used to determine the composition of the white region and dark region appeared in the backscattered SEM micrograph. The white regions A2 (white) observed in Figure 7 seem to disappear for a bond made at 20 min. This region is rich in Zn. Therefore, with more bonding time, Zn diffused away from the joint region. The C2 (dark) region that is rich in Mg looks like the A3 (dark) in terms of composition except that more Al is present for a bond made at 20 min. In the Ti side, the D1 (white) region is rich in Ti but has significantly more Zn compared to the B1 (white) region. This indicates that, at a longer time, more mutual diffusion between Ti and Zn occurs. The D2 (dark) is a rich Mg region present at the Ti fractured surface and it showed more Mg and less Zn compared to B2 (dark). Figure 9 and the corresponding elemental composition shown in Table 3 reveals similar information for a bond made at 30 min. When comparing the elemental compositions for the various regions in Figure 9 with Figure 8, it can be seen that there is no significant difference among them. The bond made at 20 min could reach the isothermal solidification stage. Therefore, no major mechanisms or changes in compositions were revealed at longer bonding times.

**Figure 10:**
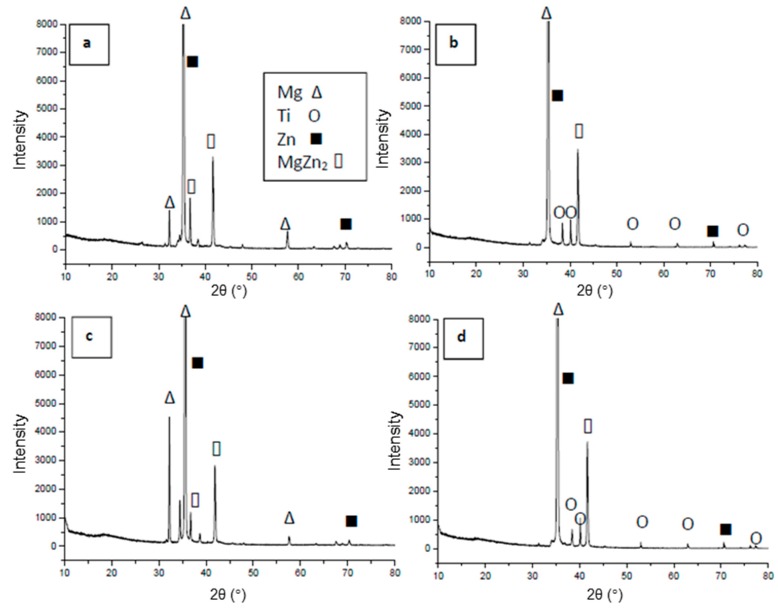
XRD patterns of the fractured surfaces for the bond made at 5 min. (**a**) Mg side and (**b**) Ti side and XRD patterns of the fractured surfaces for the bond made at 30 min. (**c**) Mg side and (**d**) Ti side.

XRD analysis was used to identify the phases formed at the fractured surfaces for bonds made at 5 min and bonds made at 30 min, as shown in Figure 10. The fractured surfaces for the magnesium side for the bond made at 5 min (Figure 10a) and the bond made at 30 min (Figure 10c) show similar patterns. High intensity peaks of Mg were observed. The patterns also showed the Zn and MgZn_2_ phases. No Ti-related peaks were observed on the Mg fractured surface. On the other hand, the titanium side of the fractured surfaces shows a strong peak of Mg, as seen in Figure 10b,d. Furthermore, the patterns from the Ti fractured surfaces show the presence of Ti, Zn, and MgZn_2_. From the XRD patterns, only MgZn_2_ IMC was detected at both fractured surfaces. No indication of IMC’s based on Ti-Zn or Ti-Al, which indicates that, at the selected bonding conditions, the only stable phase that can be formed is the MgZn_2_. The XRD patterns agreed with the SEM/EDS observations from the fractured surface, which reveal a considerable amount of Mg at both fractured surfaces. There is no significant difference and no new compounds detected by XRD through fractures at the bond made at 5 min and fracture for the bond made at 30 min. The mechanism of the bonds was not changed with changing bonding time except the change in concentrations of the elements composing the joints where the fracture occurs. Although Al was detected by SEM/EDS in a considerably noticeable amount, Al did not form IMC’s at the joint and, therefore, it could be present as a solute in the Mg-Zn eutectic and at the Mg-rich phase, which aligned with previous studies [28]. XRD analysis did not detect Al or Al-related IMC’s.

## 5. Shear Strength and Micro-Hardness Measurements

**Figure 11:**
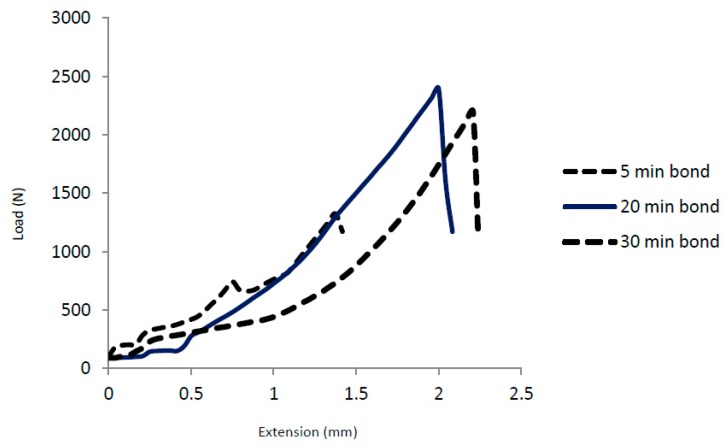
Load vs extension for the shear test of the 5-, 20-, and 30-min bonds.

The shear test was conducted for the bonds made at various bonding times. Table 4 shows the maximum load and maximum shear strength applied against each bond at the fracture point. There is an increase of the shear strength with the increase of bonding time from the bond made at 5 min to the bond made at 20 min where the maximum strength achieved was 30.5 MPa. On the other hand, when bonding time increases for more than 20 min, a slight decrease of shear strength was noticed. The optimum measured shear strength among all bonds was seen to be related to the bond made at 20 min. The load vs. extension was plotted for three bonds as seen in Figure 11 to reveal the nature of the fracture. The shear tests graph shown in the figure indicate that elongation (extension) occurs before the fracture, which means that the fracture is a ductile fracture. The extension before the fracture was measured to be 1.4 mm, 1.9 mm, and 2.25 mm for 5, 20, and 30 min bonds, respectively. The ductile fracture observed in the shear test measurements could be more evidence for the nature of the material at the joint where the fracture propagates. IMC’s are usually brittle in nature while solid solution is ductile in nature. Since microstructural analysis along with XRD analysis showed that, the joint regions mainly consist of solid solutions of Mg, Ti, Zn and Al with no major formation of IMC’s, the fracture is expected to propagate along the grains of the solid solution. The fracture occurs within the joint region mainly occupied by Mg and Zn where some Ti was detected in the joint region due to the diffusion of Ti into Zn and into MgZn. Therefore, the mechanisms of joining starts with Mg-Zn eutectic formation followed by diffusion of Zn into the Mg side and little diffusion of Zn into the Ti side. This process coincides with the diffusion/dissolution of both Mg and Ti into the joint region in order to form a solid solution.

The eutectic reaction between Mg and Zn occurs at 340 °C at the Mg-rich region (~68 at% Mg), and also occurs at 419.5 °C at the Zn-rich region (~92 at% Zn). A two eutectic points that are well below our selected bonding temperature (500 °C) would highly speed the process of TLP bonding. TLP bonding usually starts with inter-diffusion of the interlayer and base materials followed by eutectic reaction and then isothermal solidification. It ends with homogenization of the joint region. Therefore, in our case, the isothermal solidification was believed to be complete for the bonds made at 20 min where less than 7 wt% of Zn was present in the Mg side of the fracture surface seen in Table 2. When comparing the Zn content of Table 2 to the Zn content in Table 3, it can be noted that little reduction of Zn was measured for the bond made at 30 min. This indicates that the bonds made at more than 20 min were in the homogenization stage of the TLP bonding, which is a stage after isothermal solidification [6]. Al was also seen to diffuse into this joint region in all bonds made, which is proven by EDS analysis in Figure 4. This observation agrees with the work done to join Mg to Ti using the spark plasma sintering process (SPS) [18]. However, Al in our research project did not contribute to the joining process by forming any noticeable IMC’s. The only detected IMC that was formed in the joint region is the MgZn_2_, which indicate that, at the given bonding conditions, this compound is the most stable due to the fast eutectic reaction between Zn and Mg. Ti was detected at the joint region, which implies that Ti diffuses into the molten Zn and then solidifies in the matrix. This is expected by studying the Ti-Zn reaction interfaces. The time and temperature for Ti diffusion in Zn were reported to be 30 min and 500 °C, respectively [24]. This is also because both Mg and Ti have very limited mutual solubility, which makes it difficult for Ti and Mg to diffuse through each other even at a bonding temperature of 500 °C.

## 6. Conclusions

This research has shown that, despite significant differences in their physical and mechanical properties, Mg-AZ31 and Ti-6Al-4V alloys can be joined by using the TLP bonding method. The screening printing process was applied for the first time in the TLP process. This process can replace other complex processes of coatings like electroplating, thermal coatings, and physical vapour deposition (PVD). At a bonding temperature of 500 °C and various bonding times of 5, 10, 15, 20, 25, and 30 min, the joints were successfully achieved. Microstructural analysis showed the presence of Mg and Ti at the joint region as well as Zn diffused away from the joint region. There was no reaction layer formed at the joint region that changed in size with the change of bonding time, which indicates that the joining process does not rely on a growth of intermetallic compound layers. The detection of Mg in a large amount compared to Zn at the joint region indicates that, after mutual melting (Mg-Zn eutectic formation), zinc diffused away where Mg diffused to the joint region and where isothermal solidification occurred. Isothermal solidification can occur because of inter-diffusion between the coating material and the base alloy at a temperature above the eutectic temperature. On the other hand, the mechanism of bonding at the Ti side relies on the diffusion of molten Zn into Ti and the dissolution/diffusion of Ti into the joint region where Zn is present. Ti will preferably diffuse into the zinc-rich region at the joint and then will solidify in the melt. Al also diffuses into the joint region and contributes to the solidified melt since the solubility of Al in both Zn and Mg is high. XRD confirms the detection of MgZn_2_ IMC. However, this IMC is not the major phase at the joint region. The shear strength analysis confirms the ductile nature of the joint and gives a maximum shear strength of 30.5 MPa for the bond made at 20 min where isothermal solidification is completed. The slight reduction of the joint strength for the 25-min and 30-min bonds could be due to the softening of the Mg alloy at the homogenization stage.

## Figures and Tables

**Table 1:** Corresponding EDS weight/atomic composition for Figure 7.

Region	Mg (wt%/at%)	Zn (wt%/at%)	Ti (wt%/at%)	Al (wt%/at%)
A1 (eutectic)	46.9/67.1	47.0/25.0	0	6.1/7.9
A2 (white)	39.9/60.8	53.7/30.4	0	6.4/8.8
A3 (dark)	87.1/93.5	10.5/4.2	0	2.4/2.3
B1 (white)	0.9/1.8	1.5/1.1	96.6/96.1	1.1/1.8
B2 (dark)	61.4/72.7	16.6/7.3	7.7/4.6	14.4/15.3

**Table 2:** Corresponding EDS weight/atomic composition for Figure 8.

Region	Mg (wt%/at%)	Zn (wt%/at%)	Ti (wt%/at%)	Al (wt%/at%)
C1 (white)	65.5/81.3	30.1/13.9	0	4.3/4.9
C2 (dark)	88.0/92.4	6.8/2.6	0	5.2/4.9
D1 (white)	2.1/4.2	13.6/10.1	84.1/85.3	0.2/0.4
D2 (dark)	72.6/81.7	4.2/3.1	13.7/8.3	3.9/4.5

**Table 3:** Corresponding EDS weight/atomic composition for Figure 9.

Region	Mg (wt%/at%)	Zn (wt%/at%)	Ti (wt%/at%)	Al (wt%/at%)
E1 (white)	62.8/76.9	27.4/12.5	0.3/0.2	9.5/10.5
E2 (dark)	85.3/89.0	5.2/2.0	0	9.5/8.9
F1 (white)	2.8/5.4	5.7/4.1	91.4/90.2	0.2/0.3
F2 (dark)	78.8/87.7	3.9/1.6	15.3/8.6	2.1/2.1

**Table 4:** Maximum load and shear strength of the bonds made at different bonding times.

Sample	5 min	10 min	15 min	20 min	25 min	30 min
Force (N)	1324	1513	1954	2396	2253	2197.6
Strength (MPa)	16.8	19.2	24.8	30.5	28.7	28.0
